# Applying metabolomics to cardiometabolic intervention studies and trials: past experiences and a roadmap for the future

**DOI:** 10.1093/ije/dyw271

**Published:** 2016-10-27

**Authors:** Naomi J Rankin, David Preiss, Paul Welsh, Naveed Sattar

**Affiliations:** 1 BHF Glasgow Cardiovascular Research Centre; 2 Glasgow Polyomics, University of Glasgow, Glasgow, UK; 3 Clinical Trials Service Unit and Epidemiological Studies Unit, University of Oxford, Oxford, UK

**Keywords:** Metabolomics, lipidomics, pharmacometabolomics, clinical trials, intervention studies, cardiovascular disease, diabetes

## Abstract

Metabolomics and lipidomics are emerging methods for detailed phenotyping of small molecules in samples. It is hoped that such data will: (i) enhance baseline prediction of patient response to pharmacotherapies (beneficial or adverse); (ii) reveal changes in metabolites shortly after initiation of therapy that may predict patient response, including adverse effects, before routine biomarkers are altered; and( iii) give new insights into mechanisms of drug action, particularly where the results of a trial of a new agent were unexpected, and thus help future drug development. In these ways, metabolomics could enhance research findings from intervention studies. This narrative review provides an overview of metabolomics and lipidomics in early clinical intervention studies for investigation of mechanisms of drug action and prediction of drug response (both desired and undesired). We highlight early examples from drug intervention studies associated with cardiometabolic disease. Despite the strengths of such studies, particularly the use of state-of-the-art technologies and advanced statistical methods, currently published studies in the metabolomics arena are largely underpowered and should be considered as hypothesis-generating. In order for metabolomics to meaningfully improve stratified medicine approaches to patient treatment, there is a need for higher quality studies, with better exploitation of biobanks from randomized clinical trials i.e. with large sample size, adjudicated outcomes, standardized procedures, validation cohorts, comparison witth routine biochemistry and both active and control/placebo arms. On the basis of this review, and based on our research experience using clinically established biomarkers, we propose steps to more speedily advance this area of research towards potential clinical impact.

Key Messages

- Metabolomics applied to clinical trial samples can help elucidate drug mode of action, but relevant cardiometabolic examples are limited by small size and suboptimal study design.
- Clinical studies can also be used to identify novel predictors of important outcomes, in the same way as high-quality observational studies.
- Metabolomics in pre-treatment samples can be tested to predict response to therapy (both desired and undesired), but current data in cardiometabolic trials are limited.
- Early intervention metabolomics may identify changes in metabolites soon after initiation of therapy, which may predict later response to therapy or outcomes (both desired and undesired) but, again, current data from cardiometabolic trials are limited.
- Future studies of cardiometabolic trials would benefit from better design (with greater exploitation of placebo-controlled study samples and adjudicated outcomes), larger sample sizes, standardization of procedures, validation of biomarkers in new cohorts and better testing of predictive abilities against current benchmarks to demonstrate real clinical utility.

## Metabolomics, lipidomics and pharmacometabolomics

Metabolomics is defined as the study of the metabolome:^1^^,^^2^ the small molecule complement of a biological system (including drug- or microbiome-related metabolites).^3^^,^^4^ Mass spectrometry (MS) and proton nuclear magnetic resonance (^1^H-NMR) spectroscopy, utilizing targeted or untargeted methods, are the most commonly used techniques.^2^^,^^5–7^ Lipidomics, a subset of metabolomics, is the study of triglycerides, sphingomyelins, phosphatidylcholines and others, using MS methods optimized for lipids.^8^^,^^9^ Epidemiologically, metabolomic methods of phenotyping serum and plasma samples in patients and populations are attractive because they provide a large number of quantitative/semi-quantitative measures relevant to current health status and future health outcomes. The metabolite profile may be influenced by genotype, individual phenotypes, different environmental factors (e.g. diet, activity, smoking, medical treatments) and differences in the microbiome.^10^ Therefore, metabolomics may provide greater insight into mechanisms of drug action, particularly in different subgroups of patients.^11^

Pharmacometabolomics, the application of metabolomics (and lipidomics) to the study and prediction of variation in drug response, is a relatively new direction.^10^^,^^12^ One potential application is that the baseline metabolite profile (prior to pharmacotherapy) can predict drug response, in terms of both efficacy and safety, helping to stratify patients most likely to benefit from therapy or helping to select dose or type of therapy.^12^^,^^13^ Alternatively, it may be possible to use on-treatment changes in metabolites, shortly after therapy has been initiated, to identify good versus poor responders or those susceptible to adverse drug reactions. These patients can then, potentially, be offered alternative therapy or offered therapy at a more appropriate dose, so-called early intervention pharmacometabolomics.^14^ For example, prediction of drug-induced liver injury (at baseline or early post-dose) is particularly relevant to clinical trials since it is estimated that 40% of drug candidates that are discontinued in the clinical trial phase are as a result of hepatotoxicity.^15^^,^^16^ The ability to use pharmacometabolomics to predict drug-induced liver injury due to variation in metabolism of paracetamol was demonstrated by Clayton *et al.*, 2009.^17^ This was the first example of pharmacometabolomics being used in humans.^12^^,^^18^

## The value of randomized trial and intervention study biobanks

The advantage of randomized controlled trials (RCT) over observational studies is that they provide unconfounded estimates of the effect of an intervention. RCTs with appropriate clinical outcomes, for example cardiovascular disease (CVD) events linked to low-density lipoprotein–cholesterol (LDL-c) lowering, are described as having the highest level of evidence for evaluating causal pathways which integrate biomarkers.^19^ Even so, many trials are susceptible to weaknesses such as small sample size (with consequent low statistical power to detect effects of the intervention) and possible bias with, for example, open-label design. Clinical trials can be designed to investigate the effect of an intervention on surrogate outcomes and metabolic pathways, or on clinically relevant endpoints. Endpoint-driven trials are typically very large and can allow the examination of treatment effects according to various baseline characteristics and, where there are unexpected adverse or beneficial outcomes, novel predictors can also be investigated. The clinical community would welcome biomarkers that can predict variation in response to therapy. The hope is that more people who would benefit could be appropriately targeted whereas those who would potentially suffer net harm would be spared such therapy: so-called stratified (or personalized) medicine.^11^^,^^18^^,^^20^ The use of a single (or a combination of) technique(s) that provides good coverage of clinically relevant measures of small molecules, such as metabolomics, is thus highly attractive. Even more so if those methods are quality controlled, validated and provide robust identifications and absolute quantitation.^21^ However, applications to patient care must be pragmatic and they must meaningfully and cost-effectively guide care to be truly impactful.

Another value of major trials is that they typically offer rich phenotyping of all participants including conventionally measured clinical biomarkers of interest. Therefore, the added information obtained through more costly or novel biomarker approaches, such as metabolomics, can be readily measured against conventional biomarkers. This provides a realistic benchmark to ensure that the novel biomarker(s) add value over and above routine measures and are cost effective.^22^^,^^23^ Aside from randomized comparisons, the availability of adjudicated outcomes, as well as prospective follow-up in trials, also provides the platform for *post hoc* observational analyses. It is with this background that researchers value stored biobanks from such large-scale clinical intervention studies to test novel biomarkers for clinical value.

## Examples of new knowledge from use of routine biomarkers in clinical intervention studies

To anticipate where metabolomics might be impactful, and to make a useful comparison with existing metabolomics data, evidence for conventional biomarker use in clinical trials (including uncontrolled intervention studies of single arms of these trials) ought to be considered.

So far, the clinical impact of routine biomarkers to predict the degree of treatment response in many fields has been relatively modest.^24^ For example, some investigators have argued that following initiation of statin therapy it might be advantageous to track what benefit the therapy is having on an individual patient’s risk profile. Although initial trial data putatively supported an early reduction in high-sensitivity C-reactive protein (hsCRP) change as a predictor of subsequent cardiovascular benefit in addition to LDL-lowering,^23^^,^^25^ further studies from our group and others refuted this notion.^26–28^ Additionally, preliminary evidence has suggested that liver function tests may predict the degree of CVD benefit by statins, but this observation remains unconfirmed.^29^ There are also suggestive trial findings of differential effects based on baseline phenotypes with other lipid-lowering agents, for example that the fibrate class of drugs lowers cardiovascular risk more in those with high triglyceride and low HDL-c, and that ezetimibe prevents cardiovascular disease more in those with diabetes than those without.^30^^,^^31^ Whether more detailed lipidomic/metabolomic phenotyping at baseline, or on-treatment, can better inform on benefits/risks of such therapies are questions of interest.

Regarding type 2 diabetes mellitus (T2DM), patients are heterogeneous in terms of weight, insulin sensitivity, beta cell function and renal and liver function. We have limited information on which baseline phenotypes predict treatment responses to a wide range of glucose-lowering drugs with very different mechanisms. Ongoing work, such as in the MASTERMIND study (interventional cross-over study),^32^ will attempt to relate baseline phenotypes (including metabolomics) to differential treatment responses to three different classes of oral hypoglycaemic agents. The potential to enrich existing diabetes trial biobanks with metabolomics also exists and is likely to be pursued, with multiple trials now reaching completion.

This article concentrates on metabolomic gains from trials (including uncontrolled intervention studies), but the utility of trial datasets or biobanks with the availability of serially recorded routine biochemistry measures from individuals with adjudicated events should not be underestimated. These have been used to inform on predictors of events or to identify patterns of disease. For example, by exploiting the availability of serial 6-monthly liver function tests from the West of Scotland Coronary Prevention Study (WOSCOPS) (observational nested case control study, *n* = 946, 4.9 years follow-up), we noted that plasma levels of the liver enzyme alanine aminotransferase (ALT) and fasting triglyceride were the only significant independent predictors of the development of T2DM. As previous data had linked ALT levels to liver fat, these serial data were amongst the first to suggest that hepatic fat accumulation may increase before diabetes development.^33^ Such repeated measure datasets and biobanks provide excellent resources to better understand the evolution of novel metabolomics pathways, with potential for linking such changes to events of interest. Similarly, trials have been used to provide more support for cardiac biomarkers, such as N-terminal-pro-B-type natriuretic peptide (NT-proBNP), as independent predictors of CVD events.^34–36^

## Potential of metabolomics in drug trials

In terms of the investigation of mechanisms of drug action or pharmacometabolomics, it is thought that changes in metabolite concentrations after drug administration may be more pronounced (amplified) than changes in the transcriptome or proteome.^37–39^

The fact that drugs are: (i) transported using metabolite transporters (particularly membrane transporters);^37^ (ii) commonly metabolized using (phase I and II) enzymes used in the metabolism of endogenous metabolites;^40^ and that they (iii) target enzymes, receptors and transporters that evolved for endogenous metabolites^11^ suggests that metabolite concentrations are important predictors of local drug concentration and therefore drug response, and understanding of the pathways involved in drug metabolism and the effect of drug metabolites on endogenous metabolism is important in understanding drug mode of action as well as factors effecting drug efficacy (ultimately concentration of active drug at the site of action) and side effects.^40^

Measurement of only a few metabolites, for example routine or emerging biochemical biomarkers, is often inadequate to interrogate drug effects, since most drugs affect multiple interconnected metabolic pathways resulting in multiple metabolic changes.^11^ The greater coverage offered by metabolomics offers research potential. For this reason, metabolomics has the potential for clinical applications, but better quality studies are required to test this notion.^41^^,^^42^

Genomics and pharmacogenomics studies have clearly demonstrated that genetics alone cannot explain all the variation in drug response.^10^ Pharmacoproteomics also has potential, but so far has been described as lagging behind genomics.^20^ Metabolomics has been described as ‘potentially superior’ to genomics since it takes into account exogenous information [environmental influences (e.g. diet/other pharmacotherapies) or interaction with the microbiome] in addition to genetic influences.^10^^,^^15^^,^^18^

## Existing examples of use of metabolomics in trials and intervention studies

We now describe early examples of the use of metabolomics and/or lipidomics to investigate the effect of cardiometabolic disease-related pharmacotherapies on metabolite profiles in trials (including uncontrolled single-arm interventional studies), or to predict variation in drug response, with some specific examples from the cardiometabolic arena (Table 1). We excluded studies investigating only drug effects on lipoprotein profiles (as previously reviewed^43–45^).

**Table 1.** Examples of application of metabolomics to intervention studies and trials

Intervention study and brief design description	Numbers	Main findings	Strengths/limitations	Method and references
**Statins (HMG-CoA reductase inhibitors; anti-hyperlipidaemic drug)**				
Trial of **SVT80 vs SVT10/EZT10** for 6 weeks Aim: to investigate effects on lipidomic profile	39 adults with dysglycaemia and CAD 20 to SVT80 19 to SVT10/EZT10	SVT80 and SVT10/EZT10 vs baseline: no significant changes in lipid mediators (eicosanoids or endocannabinoids), ↓ global structural lipid classes, particularly SM & Cer (SM/Cer: PC ratio may be associated with ↓ risk of CVD) ↓ PC (15:0/18:2) and HexCer (d18:1/24:0) ↓ CE ↑ LysoPC 20:4, may have key role in plaque inflammation and vulnerability SVT80 vs SVT10/EZT10: ↑ LysoPC Authors postulated these molecules may have a key role in plaque inflammation and vulnerability	Randomized trial design Adjusted for FDR Correlations with TC, LDL-c, HDL-c and TG noted ○ Only free (not esterified) lipids detected	Targeted LC-MS/MS lipidomics, Snowden *et al.*, 2014

3-way cross-over trial of **RVT10, RVT40** or placebo for 5 weeks Aim: to investigate effects on lipidomic profile	12 men with metabolic syndrome	RVT10 & RVT40 vs placebo ↓ total sphingolipids (cer, SM, monohexyosylceramide, dihexosylceramide, trihexosylceramide and GM_3_ gangliocide) and LYPC, alkyl-PC, PC, alkenyl-PC, alkyphosphatidylethanolamine, alkenylphosphatidylethanolamine, phosphatidylglycerol and phosphatidylinositol Generally greater ↓ observed in RVT40 vs RVT10 These changes were independent of LDL-c/apoB-100 ↓ achieved Authors postulated ↓ SM/cer may be associated with ↓ risk of CVD	Cross-over design Adjusted for multiple comparisons Adjusted for change in LDL-c and ApoB-100	LC/MS/MS lipidomics, Ng *et al.*, 2014

Single-arm study of effect of **RVT20** for 3 weeks Aim: to investigate effects on lipidomics profile	32 healthy men	RVT20 vs baseline: ↓ SM(d18:1/16:0), SM(d18:1/18:0), TG(52:3), TG(54:4), TG(50:2), PI(36:4), PI(38:4), PE(36:2), LYPC(16:0), LYPC(18:0), PC(36:4), PC(34:2), PC(36:3), PC(40:6), PC(32:0), PC(34:1), PC(36:2) ↑ FA(20:0), LYPC(20:4), LYPC(18:1), PC(36:5), PC(38:6), PC(32:1), PC(38:5), PC(38:4), PC(36:1) The clinical significance of these changes in terms of RVT MoA is not known	○No control arm ○Healthy subjects only ○Not adjusted for FDR ○Not adjusted for ↓ in TC or LDL-c, although measures available ○Magnitude of change in lipids not reported	UPLC-QTOF lipidomics, Choi *et al.*, 2014

Single-arm study of effect of **AVT20**, single dose Aim: to predict response to single dose of AVT	48 healthy men	Low baseline concentrations of alanine, gamma-tocopherol, citric acid and arachidonic acid correlated with high area under the curve for atorvastatin Competition of metabolites and atorvastatin for monocarboxylate transporter 10 (MCT 10), organic anion transporting polypeptide 1B1 (OATP1B1) is hypothesised to explain the correlation of the baseline metabolites and AUC of atorvastatin	Diet and other exogenous influences minimized Internal model validation ○Some routine biochemistry measures included, not adjusted for ○No control arm ○Healthy subjects only ○Not adjusted for FDR ○Not adjusted for ↓ in TC or LDL-c ○Single dose	GC-MS metabolomics, Huang *et al.*, 2015

Single-arm 6-week non-randomized trial of **SVT40** in African-American and Caucasian men and women Aim: to investigate effects on lipidomic profile and bile acids and to relate pre- and post-SVT profiles to variation in LDL-c lowering	148 individuals’ samples analysed from larger study: 24 GRs (based on change in LDL-c); 24 PRs; and 100 randomly selected individuals	Baseline CE and PL metabolites, particularly ratio of 20:4n6 to 20:3n6, correlated with change in LDL-c Authors postulate this indicates ↑ desaturase activity resulting in ↑ eicosaonoids (via 20:4n6) Authors postulate variation in plasmologen metabolism may influence the anti-inflammatory effects of SVT	○ No control arm ○ No correction for FDR ○ Magnitude of change in lipids or bile acids not reported ○ Surrogate marker study	Targeted GC lipidomics, Krauss *et al.*, 2013
		Baseline concentrations of four bacterially derived bile acids/sterols predicted SVT response Plasma concentrations of several bile acids were correlated with SVT concentration; they share the same hepatic/intestinal transporter		Targeted GC-MS method for sterols and bile acids, Krauss *et al.*, 2013
		↓ baseline concentrations of xanthine predicted GR. Authors postulate this results in ↑ in nitric oxide synthase (NOS) activity (via which statins improve endothelial function) SVT therapy ↑ AA degradation AA concentrations correlated with LDL-c change; again, authors postulate this results in ↑ NOS ↓ baseline levels of 2-hydroxyvaleric acid predicted GR; authors postulate this indicates ↓ bacterial enzyme activity resulting in ↓ SVT degradation SVT ↓ 2-hydroxyvaleric acid; again implicating ↓ SVT degradation as above In GR: SVT ↑ shikimic acid, a bacterial metabolite, again highlights the potential importance of microbiome		Untargeted GC-ToF-MS, Krauss *et al.*, 2013

Single-arm 6-week non-randomized trial of **SVT40** in African-American and Caucasian men and women Aim: to investigate effects on metabolite profile and relate pre- and post-SVT profiles to variation in LDL-c lowering	148 individuals’ samples analysed from larger study: 24 GRs (based on change in LDL-c); 24 PRs; and 100 randomly selected individuals	↓ baseline concentrations of uridine and pseudouridine predicted the greatest ↓ in LDL-c ↓ baseline concentration of xanthine, 2-hydroxyvaleric acid, succinic acid and steric acid with ↑ baseline galactaric acid were correlated with GR. These metabolites, alongside others, were used to build a robust model that could predict response to statin SVT40 vs baseline: ↓ cholesterol, α and γ-tocopherol and lauric acid and ↑ threonine, alanine and phenylalanine indicating ↑ in AA degradation ↑ shikimic acid was observed in GRs, a bacterial metabolite, indicating ↑ in microbial synthesis and/or ↑ in intestinal absorption (via transporters). In PR ↓ in glucose, fructose and glycolic acid were observed Changes in urea cycle metabolites and dibasic AAs correlated with change in LDL-c Authors postulate pleiotropic effects of SVT influence SVT response in terms of LDL-c lowering	Corrected for FDR ○ No control arm ○ Magnitude of change in lipids or bile acids not reported ○ Surrogate marker study	Untargeted GC-ToF-MS, Trupp *et al.*, 2012

Single-arm 6-week non-randomized trial of **SVT40** in African-American and Caucasian men and women Aim: to identify baseline metabolites that can predict LDL-c lowering	148 individuals’ samples analysed from larger study: 24 GRs (based on change in LDL-c); 24 PRs; and 100 randomly selected individuals	↓ baseline concentrations of five 1^a^ and 2^a^ bile acids (TCA, GCA, TCDCA, GCDCA, GUDCA and TDCA) were correlated with greater ↓ in LDL-c after SVT in 100 randomly selected samples ↑ baseline concentrations of 3 2^a^ bile acids (LCA, TLCA and GLCA) and the enterically produced sterol COPR were correlated with greater ↓ in LDL-c after SVT in 48 PR vs GRs Genotyping of an SNP in an organic anion transporter demonstrated associations with bile acid concentration Demonstrates potential utility of metabolomics in identifying predictors of GR vs PR Highlights role of microbiome in modulating SVT blood concentration and therefore effect	○ No control arm ○ Not corrected for FDR ○ Magnitude of change in bile acids not reported ○ Surrogate marker study	Targeted GC-MS metabolomics, Kaddurah-Daouk *et al.*, 2011

Cross-over trial of **SVT40** vs placebo for 2 weeks Aim: to investigate the effect on lipidomics profile	29 men with mixed dyslipidaemia 15 SVT40 then placebo 14 placebo then SVT40	SVT40 vs placebo: ↓ FC, CE, TG, PE and LY Out of 33 FAs evaluated, 9 were ↓ after SVT40 However this was not observed in the 5/29 men who did not respond to SVT40 (same/↑ LDL-c observed, despite compliance) Concentrations of most abundant fatty acids correlated with LDL-c and TG, but not HDL-c Authors suggest the ↓ in lipid classes observed are due to ↑ clearance of LDL/IDL and VLDL particles. However there may also be differential metabolism Conversely, lack of ↓ in lipid classes may be due to lack of ↓ in LDL/IDL or VLD particles	Randomized trial design Controlled for FDR	Capillary GC-FID lipidomics, Chen *et al.*, 2011

Single-arm 6-week non-randomized trial of **SVT40** in African-American and Caucasian men and women Aim: to investigate effects on metabolite profile and relate pre- and post-SVT profiles to variation in LDL-c lowering	48 individuals’ samples analysed from larger study: 24 GRs (based on change in LDL-c); 24 PRs	Baseline concentrations of 7 lipids, particularly ω-3 and ω-6 lipids, were positively correlated with ↓ in LDL-c Baseline concentrations of 8 lipids, particularly PE plasmologens and PC plasmologens, were correlated with ↓ in CRP; these did not overlap with lipids that correlated with LDL-c response On-Rx GRs: ↓ TG, CE, FC, PC and PE. Many FA in CE, DG, LY, PC, PE and TG ↓ 2 FAs ↑ (20:1n9 and 20:3n3) and 2 LYs ↑ (20:4n6 and 20:5n3) On-Rx PRs: ↓ TG, fewer ↓ observed in all classes, 5 ↑ observed Larger ↓ in lipids correlated with greater ↓ LDL-c Few changes in lipids correlated with ↓ CRP Demonstrates potential utility of metabolomics in identifying predictors of GR vs PR	Corrected for FDR ○ No control arm ○ Surrogate marker study	GC-FID lipidomics, Kaddurah-Daouk *et al.*, 2010

Randomized trial of **RVT vs AVT** for 18 weeks Aim: to investigate effects on metabolite profile and relate pre- and post-dose profiles to variation in LDL-c lowering	80 adults 39 randomized to either RVT10, RVT20 and RVT40 41 to AVT20, AVT40 and AVT80 (6 weeks at each dose)	PLS-DA showed lipidomic profile could be used to differentiate RVT vs AVT-treated patients. These were predictive of lowering of LDL-c:HDL-c ratio SM and CE were particularly important in predicting ↓ in LDL-c:HDL-c ratio RVT vs AVT: ↑ PC (36:4) and PC (38:4) RVT vs AVT: greater ↓ in SM (18:0) & lesser ↓ in ratio of SM:(SM&PC) Demonstrates RVT and AVT have different effects on lipidomic profile, which may contribute to variation in potency/effect of different statins	Randomized design Multivariate analysis chosen to minimize FDR ○ Magnitude of change in lipids not reported ○ Surrogate marker study	HPLC/MS lipidomics, Bergheanu *et al.*, 2008

Randomized trial of **SVT80, AVT40** or placebo for 8 weeks Aim: to identify biomarkers of myotoxicity (early post-dose) and elucidate mechanism of myotoxicity	37 adults 11 to placebo 12 to SVT80 14 to AVT40	PLS-DA showed SVT, AVT and placebo had different effects on lipidomic profiles. Several PEs and LCTGs were ↑ in SVT AVT and several PCs and CE were ↓ Combined with gene expression analysis of muscle biopsy: lipidomic changes correlated with arachidonate 5-lipoxygenase-activating protein gene expression in muscle tissue Authors describe their combined lipidomics/transcriptomics platform as an early sensitive marker of statin induced metabolic changes in muscle; however, no patient in the study developed ↑ CK or complained of muscle symptoms during the study; they were not followed up to see if they did develop muscle symptoms	Randomized design ○ Not corrected for FDR ○ CK measured but not reported; no patient developed raised CK during the study ○ No cases of muscle myopathy so clinical utility is unknown ○ Magnitude of change in lipids not reported	UPLC-MS lipidomics, Laaksonen *et al.*, 2006

**Fenofibrate (FFB)**				
Subset of randomized trial of **FFB** (200 mg/day) vs placebo in patients with T2DM for 5 years (with ↓ vs ↑ Hcy on Rx) Aim: to investigate effect on HDL lipidomic profile in individuals who had ↓ vs ↑ Hcy on Rx	47 adults with T2DM 17 on FFB with ↓ Hcy 16 on FFB with ↑ Hcy 14 on placebo with ↓ Hcy	FFB in both groups vs placebo: ↑ SM-rich signal transduction and membrane lipids, ↑ PC-rich membrane and ether-linked lipids, ↓ LYPC FFB in ↓ Hcy group only vs placebo: ↑ ether-PL Demonstrates change in HDL lipidomics profile differs in those with ↓ vs ↑ Hcy on Rx Authors postulated combination of HDL lipidomics profile and molecular dynamics could identify surrogates for predictors of drug response in the future	Modelling method chosen to minimize FDR ○Surrogate marker study	UPLC-MS lipidomics of HDL subfractions, Yetukuri *et al.*, 2011^103^

Single-arm 2-week study of 200 mg/day **FFB** Aim: to identify urinary biomarkers of PPARα activation	10 healthy volunteers	FFB vs baseline: ↓ pantothenic acid, acetylcarnitine, propylcarnitine, isobutyrylcarnitine, (s)-(+)-2-methylbutyrylcarnitine and isovalerylcarnitine Highlights the potential of metabolomics in aiding understanding of drug MoA and variation in drug response Discriminating metabolites were confirmed using authentic compounds where possible Discriminating metabolites were quantified by specific assay Biomarkers confirmed in animal study wild-type vs PPARα-null mice	Corrected for FDR Routine biomarkers reported and compared ○Healthy volunteers may not reflect MoA in disease group	UPLC-MS of urine, Patterson *et al.*, 2009

**Anti-hypertensive therapies: beta blocker (atenolol)**				
Nested case-control study of single-arm RCT of **atenolol** for 9 weeks Aim: to determine if AA profile can predict IFG post-atenolol	122 European-Americans with mild-moderate essential hypertension on atenolol 24 developed IFG 98 did not	↑ baseline concentrations of four amino acids (Isoleucine, leucine, valine and phenylalanine) found to predict development of IFG in atenolol-treated adults. Model adjusted for age, sex, BMI, fasting glucose, insulin and HOMA-IR Combination with genotypes for 2 enzymes involved in AA catabolism identified SNPs in phenylalanine hydroxylase associated with ↑ risk of IFG However, as there was no control arm in this study, it is not possible to determine if the model predicts atenolol-induced IFG or risk of IFG without a pharmacological/other trigger (atenolol known to ↑ risk of IFG)	Prospective Adjusted for baseline glucose, insulin, HOMA-IR etc ○ No control arm	Targeted ToF MS amino acid analysis, Cooper-DeHoff *et al.*, 2014

*Post hoc* study of single-arm RCT of **atenolol** for 9 weeks Aim: to determine effect on metabolomic/lipidomic profile and relate this to racial variation in response to atenolol	272 patients randomly selected from each quartile of BP response 150 Caucasians 122 African Americans	Caucasians vs African Americans: ↑ in the effect of atenolol on BP and renin activity ↓ in palmitic, oleic, palmitoleic, arachidonic and linoleic acid and 3OHB Combined with geneotyping of lipase genes: race-specific associations between SNPs and Fas found Demonstrates potential of pharmacometabolomics in understanding variability in response to atenolol based on race and genotype	Controlled for FDR Compared with routine measures, only plasma renin differentiated Caucasians from African Americans ○ No control arm ○ More females in African American arm	GC-ToF-MS, Wikoff *et al.*, 2013

**Anti-platelet therapy: Ximelagatran (oral anti-coagulant) and aspirin**				
*Post hoc* study of single-arm RCT of **ximelagatran** for 17 months Aim: to identify biomarkers that can predict ↑ ALT	134 participants with AF 34 cases with ALT 3-9*ULN; 12 cases ALT >9*ULN 86 controls	Pre-dose samples identified formate, cystine, creatinine, glutaminc acid, pyruvic acid, alanine, 2-ketoglutaric acid as putative biomarkers for ALT elevation Ximelagatran Rx resulted in changes in 3OHB, pyruvic acid, glutamine, vitamin E, phenylalanine, tyrosine, a number or monoglycerides and triglycerides Highlights potential of metabolomics in prediction of drug induced liver injury and in understanding MoA in terms of toxic side effects	Corrected for FDR Combined with proteomics Hepatocytes cultured with various concentrations of 2 metabolites identified as predicting ↑ ALT ○ No control arm ○ Lack of time point-matched samples (can result in confounding) ○ Surrogate marker study	LC/MS/MS, GC-MS and ^1^H-NMR, Andersson *et al.*, 2009

Single-arm study of **aspirin** intervention (81 mg/day) for 2 weeks Aim: to investigate the effect on metabolite profile and identify novel mechanisms of aspirin resistance	76 healthy Amish volunteers 40 GRs 36 PRs (as determined by collagen stimulated platelet aggregation *ex vivo*)	18 metabolites were found to be significantly altered by aspirin Rx, 2 were aspirin catabolites (salicylic and salicyluric acid), 6 were metabolites of purine metabolism. Of these, inosine and adenosine were ↑ in PRs compared with GRs Guanosine, hypoxanthine and xanthine were also altered after Rx, with potential effects on aggregation and CVD risk Results were replicated in another 37 participants (19 GR and 18 PRs) Pharmacogenomics identified an SNP in adenosine kinase which was associated with purine metabolism and aspirin response Highlights potential of metabolomics in understanding drug MoA Highlights potential of pharmacometabolomics in early prediction of GR vs PR	Corrected for FDR Pharmacometabolomics informed pharmacogenomics Replicated in another 49 participants and 341 participants from a similar study ○ No control arm ○ Healthy volunteers may not reflect mechanisms in those with CAD ○ Magnitude of change in metabolites not reported ○ Surrogate marker study	Untargeted GC-MS, Yerges-Armstrong *et al.*, 2013

Single-arm study of **aspirin** intervention (81 mg/day) for 2 weeks Aim: to investigate the effect on metabolite profile and investigate mechanisms of variation in aspirin response	80 healthy Amish volunteers 42 GRs 38 PRs (as determined by collagen stimulated platelet aggregation *ex vivo*)	19 out of the 35 metabolites measured were significantly altered post Rx compared with baseline Metabolites were different in GR vs PR In particular, baseline serotonin levels were ↑ in PRs and ↑ further after Rx in PRs Many of these differences were replicated in a validation study of 125 individuals Highlights potential of pharmacometabolomics in baseline/early prediction of GR vs PR Effect of serotonin on coagulation pre- and post-aspirin confirmed *ex vivo*	Corrected for FDR Replicated in another 125 participants ○ No control arm ○ Healthy volunteers may not reflect mechanisms in those with CAD ○ Magnitude of change in metabolites not reported ○ Surrogate marker study	Targeted analysis of 1^a^ and 2^a^ amines by UPLC-MS, Ellero-Simatos *et al.*, 2014

**Anti-angina therapy: perhexiline**				
Randomized trial of biopsies of left ventricular wall taken during CABG after ≥ 5 days placebo vs oral **perhexiline** Aim: investigate effect on myocardial metabolite profile	43 biopsies were analysed 22 perhexiline-treated patients 21 controls (placebo)	Oral perhexiline did not provide myocardial protection No significant effect on the myocardial metabolome was observed. Authors postulate this supports the suggestion that it is not acting on myocardial pathways dependent on myocardial CPT-1 inhibition and perhaps explains the lack of clinical benefit observed	Randomized trial design Placebo controlled Prospective Corrected for FDR No significant changes in troponin-T either ○ Surrogate marker study ○ Magnitude of change in metabolites not reported	FT-ICR-MS, Drury *et al.*, 2015

**Insulin sensitizing agent: rosiglitazone**				
Randomized trial of **rosiglitazone** vs placebo for 16 weeks Aim: to investigate the effect on metabolite profile and relate these to changes in myocardial glucose uptake	51 adults with T2DM and CHD 25 to rosiglitazone (4-8 mg) 26 to placebo	Rosiglitazone vs placebo: ↑ glutamine and ↓ lactate Reflects improved insulin sensitivity ↓ lactate correlated with ↑ myocardial glucose uptake	Randomized trial design Placebo controlled Corrected for FDR ○ Not compared with routine measures ○ Surrogate marker study	^1^H-NMR metabolomics, Badeau *et al.*, 2014^104^

Randomized trial of **rosiglitazone** vs **metformin** vs **repaglinide** for 48 weeks Aim: to investigate the effect of drug therapy metabolite profile	82 adults newly diagnosed with T2DM 25 to rosiglitazone 22 to metformin 35 to repaglinide plus	All three: ↓ glutamate Rosiglitazone: ↓ valine, lysine, glucuronolactone, C16:0, C18:1, urate and octadecanoate Metformin and repaglinide did not significantly improve the metabolic profiles	○ Randomized trial design ○ Compared with routine measures ○ Not corrected for FDR ○ Surrogate marker study	GC-MS metabolomics, Bao *et al.*, 2009

Randomized trial of 8 mg/day **rosiglitazone** vs placebo for 6 weeks Aim: to investigate the effect on metabolite profile	32 adults 16 individuals with T2DM 16 healthy volunteers	Urine In T2DM individuals on RSG vs placebo: ↓ urinary hippurate and ↑ urinary AAA In healthy controls on RSG vs placebo: no changes in urinary metabolite profile Plasma In T2DM males on RSG vs placebo: ↑ BCAA, alanine, glutamine/ glutamate and threonine In T2DM females on RSG vs placebo: ↑ BCAA, alanine, glutamine /glutamate and citrate with ↓ lactate, acetate, tyrosine and phenylalanine In healthy controls on RSG vs placebo: no changes in plasma metabolite profile Demonstrates potential of metabolomics in understanding drug MoA	Randomized trial design Placebo controlled ○ Age gap between T2DM and healthy volunteers ○ Not corrected for FDR ○ Changes in routine measures not reported ○ Magnitude of change in metabolites not reported	^1^H-NMR metabolomics of plasma and urine, Van Doorn *et al.*, 2007

**Anti-hyperglycaemic drug: Metformin**				
Comparison of adults without T2DM treated with **metformin** vs placebo for 18 months Aim: to investigate the effect of metformin on amino acids	173 adults without T2DM but with coronary disease 86 to metformin 87 to placebo	Metformin vs placebo: ↓ tyrosine and phenylalanine; with ↑ alanine and histidine Concentrations of leucine, isoleucine valine and glutamine did not significantly differ Concentrations of lactate and pyruvate did not significantly differ	Randomized trial design Placebo controlled Adjusted for insulin resistance etc ○ No correction for FDR ○ Surrogate marker study ○ Individuals did not have T2DM (by design)	Targeted NMR metabolomics of plasma, Preiss *et al.*, 2016

Comparison of adults with IFG or untreated DM treated with **metformin and piogitazone** for 3 months vs placebo Aim: to investigate the effect of metformin and pioglitazone on AAs compared with placebo	25 overweight/obese adults with IFG or untreated DM 12 to metformin and pioglitazone 13 to placebo	Metformin and pioglitazone combination therapy reduced 9 out of 33 AAs and AA metabolites (phenylalanine, tyrosine, citrulline, arginine, lysine, α-aminoadipic acid, aspartic acid, glutamic acid and ethanolamine)	•Randomized trial design •Placebo controlled ○ Combination therapy ○ Not adjusted for FDR	Targeted AA analysis using LC-MS/MS, Irving *et al.*, 2015^105^

Comparison of adults with T2DM treated with **metformin** for 3 years vs glipizide Aim: to investigate the effect of metformin on lipids compared with glipizide	44 adults with T2DM and CAD 23 to metformin 21 to glipizide	12 lipids (LPC (16:1), LPC (18:1), LPC (20:3), LPC (20:4), LPC (22:6), PC (34:0), PC (O-34:2), PC (O-36:4), PE (36:4), PE (38:6), SM (d18:1-14:0), and SM (d18:1-16:1) were significantly different between metformin- and glipizide-treated participants, three of these were associated with CVD endpoint Increase in TAG acyl chain carbon number and slight increase in TAG with 0 to 3 double bonds was observed in the metformin-treated group compared with the glipizide-treated group Although the association between these changes and CVD risk is unclear, these changes may help explain the protective effect of metformin in CVD	• Endpoint = CVD events • Randomized trial design • Significant changes in routine biochemistry measures (glucose, HbA1c and lipids) were not observed ○Not adjusted for FDR	LC-MS lipidomics, Zhang *et al.*, 2014

Comparison of adults with T2DM treated with **metformin** for 3 months vs untreated controls Aim: to investigate the effect on metabolite profile	35 adults with T2DM 20 on no treatment 15 on metformin	Metformin vs no Rx: ↓ glucose, N-acetyl glycoprotein, lactate, acetoacetate, lysophosphatidylcholines (16:0, 18:0 and 18:2) and phenylalanine ↑ TMAO, 3OHB and tryptophan Demonstrates potential of metabolomics in understanding drug MoA	○ Not placebo controlled ○ No correction for FDR ○ Routine measures not reported ○ Magnitude of change in metabolites not reported	^1^H-NMR metabolomics and UPLC/MS, Huo *et al.*, 2009

Diclofenac (non-steroidal anti-inflammatory)				
Randomized trial of 150 mg/day **diclofenac** vs placebo for 9 consecutive days Aim: to investigate the effect of modulation of obesity-associated inflammation using diclofenac on metabolite profile	19 overweight males (BMI 25-31 kg/m^2^) 9 randomized to diclofenac 10 randomized to placebo	Diclofenac vs placebo: 19 oxolipids found to vary. However, many of these correlated with CRP ↑ 20-HETE, 5,6-DHET and ↓ 9,10-DHOME were independent of CRP change Demonstrated potential of metabolomics in identifying markers of modulation of inflammatory response using drug therapy	Randomized trial design Placebo controlled Corrected for model over-fitting Correlations with CRP investigated Integration with transcriptomics of peripheral blood mononuclear cells and proteomics	LC-MS and GC-MS, Van Erk *et al.*, 2010

**Abbreviations:** 1^a^, primary; 2^a^, secondary; 3OHB, 3-hydroxybutyrate; 20-HETE, 20-hydroxyeicosatetraenoic acid; 5,6-DHET, 5,6-dihydroxy-eicosatrienoic acid; 9,10-DHOME, 9,10-dihydroxyoctadecenoic acid; ω-3 and ω-6, omega 3 and 6; AA, amino acid; AAA, aromatic amino acid; ADR, adverse drug response; AF, atrial fibrillation; ALT, alanine transaminase; ApoB-100, apolipoprotein B 100; AVTnn, atorvastatin (nn mg/day); BCAA, branched chain amino acid; BMI, body mass index; BP, blood pressure; CABG, coronary artery bypass graft; CAD, coronary artery disease; CE, cholesterol ester; cer, cerimide; CHD, coronary heart disease; CK, creatinine kinase; COPR, coprostanol; CRP, C-reactive protein; CVD, cardiovascular disease; DG, diacylglycerol; EZT, ezetimibe; FA, fatty acid; FC, free cholesterol; FDR, false discovery rate; FFB, fenofibrate; FID, flame ionization detector; FT-ICR-MS, fourier transform ion cyclotron resonance mass spectrometry; GC, gas chromatography; GCA, glycocholic acid; GCDCA, glycochenodeoxycholic acid; GLC, glycolithocholic acid; GM3 gangliocide, monosialodihexosylgangliocide; GR, good responder; GUDCA, glycoursodeoxycholic acid; Hcy, homocysteine; HDL-c, high-density lipoprotein-cholesterol; HexCer, hexosyl-ceramide; HPLC, high performance liquid chromatography; HOMA-IR, homeostatic model assessment insulin resistance; IFG, impaired fasting glycaemia; LC, liquid chromatography; LCA, lithocholic acid; LC-MS/MS, liquid chromatography tandem mass spectrometry; LCTG, long-chain triglyceride; LDL-c, low-density lipoprotein-cholesterol; LYPC, lysophosphatidylcholine; MoA, mechanism of action; MS, mass spectrometry; ^1^H-NMR, nuclear magnetic resonance; nn, number; NO, nitric oxide synthase; PC, phosphatidylcholine; PE, phosphatidylethanolamine; PI, phosphatidylinositol; PL, phospholipid; PLS-DA, partial least squares discriminant analysis; PPAR, peroxisome proliferator-activated receptor; PR, poor responder; RCT, randomized controlled trial; RSG, rosiglitazone; RVTnn, rosuvastatin (nn mg/day); Rx, treatment; SM, sphingomyelin; SNP, single nucleotide polymorphism; SVTnn, simvastatin (nn mg/day); T2DM, type 2 diabetes mellitus; TC, total cholesterol; TCA, taurocholic acid; TCDCA, taurochenodeoxycholic acid; TDCA, taurodeoxycholic acid; TG, triglyceride; TLCA, taurolithocholic acid; TMAO, trimethylamine N-oxide; ToF, time of flight; ULN, upper limit of normal; UPLC/QTOF/MS, ultra-performance liquid chromatography quadrupole time of flight mass spectrometry.

### Strengths of metabolomic studies in trials to date

All of the studies used state-of-the art analytical methods (Table 1). Many of the studies investigating pharmacometabolomics or effects of pharmacotherapies on the metabolome were well designed and included randomization and control arms (Table 1). Many included important endpoints (such as LDL-c lowering after statin therapy); however, only one had a hard endpoint (CVD outcome in glipizide-treated patients).^46^ A number of studies included routinely measured biomarkers for comparison.^46–55^ The majority of studies also adjusted for multiple statistical comparisons (Table 1). Some performed internal model validation^55^^,^^56^ and two studies performed external model validation.^57^^,^^58^ Some studies also combined metabolomics data with genomic and proteomic data.^55^^,^^57^^,^^59^

### Hypothesis-generating/*post hoc* studies of the effect of cardiometabolic drugs on metabolomics biomarkers

The majority of studies that have measured metabolomics in samples from clinical intervention studies are the result of *post hoc* or hypothesis-generating studies (Table 1), as often recognized by the authors themselves. In most cases, published data to date are from studies that are small in size (the largest being a study of 272 adults^51^), consequently they may be limited in power. Despite this some have shown some promise for the future of metabolomics, and the clear need for hypothesis-generating research has been highlighted.^60^^,^^61^ However, we also need to acknowledge that a higher standard of research is now required to advance the field to a degree where real-world clinical applications of metabolomics can be considered. We therefore highlight examples of weaknesses in existing research that might be improved in the future.

Most studies have investigated how a specific drug may alter metabolic profile (mode of actions studies), but little else (Table 1). Only a few have linked any specific metabolite to clinical outcomes, or to prediction of benefit or harm, primarily due to lack of power. Often only surrogate markers for clinical outcomes were used: LDL-c lowering as a marker for statin-related CVD reduction,^62–65^ ALT as a marker for liver injury,^59^ platelet aggregation as a marker for anti-platelet effects^57^^,^^58^ or low cardiac output as a marker for myocardial protection,^52^ for example. Whereas these studies are of interest, they are currently limited in terms of clinical translation.^66^

Few studies compared metabolite-based biomarkers with routine measures (Table 1).^47–52^^,^^55^ For example, in a lipidomic study of rosuvastatin’s effect in healthy volunteers, a number of lipids were found to be decreased.^8^ Some decreases will be entirely dependent on increased clearance of LDL particles as a result of rosuvastatin therapy and therefore, in isolation, such data provide limited additional insight into mechanisms of rousuvastatin or clinical implications of treatment. In a previous lipidomics study of the effect of simvastatin in men with mixed dyslipidaemia, reductions in fatty acids (FAs) were associated with increased clearance of LDL particles; no reductions in FAs were observed in non-responders who did not have a decrease in LDL-c.^67^ Again, such findings are hypothesis-generating and require further detailed and robust study.

Approximately half of included studies were *post hoc* analysis of interventional studies used for observational investigations (no longer placebo-controlled) or are based on analysis of single-arm interventional studies (Table 1).^8^^,^^49–51^^,^^57–59^^,^^62–65^ Although they are included in this narrative review, it should be noted that evidence from such studies is potentially biased or confounded. For example, in an investigation of the effect of rosuvastatin on lipidomic profile, changes were compared with baseline concentration only,^8^ and in a study of metformin’s effect on metabolite profile, metformin-treated individuals were compared with individuals with no therapy.^68^ Atenolol is a commonly used selective adrenergic B1 receptor antagonist that is known to increase the risk of hyperglycaemia, impaired fasting glycaemia (IFG) and diabetes mellitus^50^ (Table 1). In a nested case-control study of the Framingham Offspring Study, a combination of five amino acids was found to modestly predict the risk of diabetes or insulin resistance.^69^ Using a hypothesis-driven approach to determine if these same amino acids predicted risk of IFG after atenolol therapy, other researchers performed amino acid analysis (described as targeted metabolomics) in 122 adults treated with atenolol for a mean of 9 weeks as part of a nested case-control study of an interventional study.^50^ They demonstrated that the same amino acids could predict individuals most likely to develop IFG after atenolol treatment. The authors suggested that treatment with drugs such as atenolol may be an environmental trigger in these at-risk individuals. However, as the study only included participants from the atenolol arm (with no control arm), it is not possible to speculate on how many individuals would have developed IFG anyway, nor whether usual predictors of diabetes would have given the same or better information on diabetes prediction.

Some studies included only healthy individuals (for example studies of fenofibrate^49^ and aspirin^57^^,^^58^). Changes in metabolite concentrations in healthy individuals may differ from changes in metabolite concentration in intended recipients of pharmacotherapy.^51^ Other studies were limited by the availability of time-matched samples. In a study of ximelagatran toxicity, samples from cases of drug-induced liver injury were not available from controls at the same time points, so causal inferences cannot be made with any confidence.^59^ In some studies, patients in different treatment arms were not well matched at baseline, for example gender imbalance in a study of atenolol in African Americans versus Caucasians,^51^ and age imbalance between healthy volunteers and patients with T2DM in a study of rosiglitazone.^70^ There is also the strong likelihood of publication bias, with negative studies being very slow to publish or left unpublished.^71^ Hence, most of the studies published to date should, at best, be described as hypothesis-generating, and few as yet have added clinically meaningful insights.

Finally, a recent observational study took advantage of a parallel genetic analysis to show almost identical changes in lipoprotein and lipidomic profiles with statin use (versus non-use) and corresponding genetic instruments (HMGCR rs12916 variant known to mimic HMGCR inhibition).^72^ No robust changes were observed for the metabolites measured (amino acids, ketones, glycolysis- and gluconeogenesis-related metabolites) in the statin-treated or the rs12916 variant groups, suggesting minimal pleiotropic effects. This study is therefore an exemplar for future combined genetic/phenotyping studies, so called ‘natural’ or *in silico* clinical trials, to determine metabolomics effects of drugs.^73^

### Examples of metabolomics studies limited by lack of clinical utility

As is the case for many drugs, there is clinical variation in the effects of statins on LDL-cholesterol. In one study, decreases in LDL-c ranged from less than 5% to more than 60% on simvastatin 40 mg daily (however, LDL-c lowering was as expected in the majority, i.e. > 30% in >  75% of participants, and > 20% in >  90% of participants) even when concordance with therapy was apparently accounted for.^65^ Investigators have therefore sought to determine whether metabolomics can help predict degree of statin efficacy in terms of LDL-cholesterol lowering (Table 1).^62–65^^,^^74^^,^^75^ Here, preliminary evidence indicates secondary bile acids and amino acids produced by the gut microbiome may contribute to the inter-individual variation in LDL-c lowering response.^62–64^ Baseline concentrations of secondary bile acids and other metabolites have been found to predict the circulating statin concentration and the degree of LDL-c lowering by statins,^62^^,^^63^^,^^65^ but more robust studies are needed to confirm these findings.

Whereas these studies are of academic interest, our view is that the use of pharmacometabolomics in prediction of statin LDL-c-lowering response in clinical practice is likely to be impractical, given the huge numbers of patients eligible for statin therapy, the low cost of treatment, the safety of statins and the LDL-c lowering that is likely to occur in the vast majority. Patients routinely start on a standard statin dose (e.g. atorvastatin 20 mg/day for primary prevention and atorvastatin 80 mg/day for secondary prevention) unless there is potential for drug interaction or there appears a high risk of adverse events.^76^ If a patient is found to have responded poorly to statin therapy, i.e. a lack of LDL-c reduction (or better non-HDL-c change) using routine biochemistry analysis,^77^ adherence and diet/lifestyle changes are discussed and the dose can be increased. It is unlikely that the current cost of predicting patient response using metabolomics, even if this were possible with a high degree of accuracy, whether at baseline or early post intervention, would be clinically effective or cost effective in this regard. Similar limitations have been outlined for the use of pharmacogenomics on the prediction of optimal statin therapy.^78^ Interestingly, the Clinical Pharmacogenetics Implementation Consortium has issued guidelines for using SLCO1B1 to inform simvastatin dose, but only when genetic data are already available.^79^ In our view, metabolomics measurements to predict response to therapy may well have a place with medicines where risk/benefit is more closely balanced, and where response to therapy is more variable. We believe that there is an important distinction to be made between cheap, safe and clinically effective pharmacotherapies such as statins and more expensive, risky pharmacotherapies that would more likely benefit from tailoring, such as oncotherapy. In such situations a strong case can be made for detailed phenotyping to allow selection of individuals most likely to benefit.

Certainly there is a variation in response to statins and this does correlate with efficacy in terms of CVD risk reduction.^80^ However, in many cases where expected response to statin therapy is not observed, poor adherence to therapy or discontinuation of therapy is the usual cause.^81^^,^^82^ Such cases will not be helped by pharmacometabolomics. It is also important to note that although on-treatment reduction in LDL-cholesterol is only one measure of statin effectiveness, it is a simple, cheap and effective measure of response (and adherence) to statin therapy.^83^

Most importantly, we note that there are currently no lipid-modifying therapies that can compete with statins in terms of both clinical and cost effectiveness. There is no evidence that a patient who does not respond well to a statin would respond better to ezetimibe or bile acid sequestrants. Withholding statin therapy based on pharmacometabolomics and treating with such alternatives would likely be unethical. New LDL-cholesterol lowering therapies such as PCSK9 inhibitors may be more widely prescribed in future (currently limited to patients with familial hypercholesterolaemia and those receiving statin therapy for secondary prevention who do not achieve LDL-c targets).^80^ These agents have to be injected, are hugely expensive and have not yet been proven to reduce CVD risk. Giving patients such therapies based on pharmacometabolomics results is again unlikely, in our view. It remains to be shown (in clinical trials) that pharmacometabolomics is superior to monitoring of LDL-cholesterol response, and we would argue that given the low costs and effectiveness of statin therapy, and the low cost of monitoring, this is unlikely to be the case.

Despite our reservations concerning pharmacometabolomics for the purpose of predicting statin efficacy, we agree that pharmacometabolomics of statin therapy may have a role in identifying potential biomarkers of myopathy (Table 1).^9^^,^^75^^,^^84^ UPLC/MS lipidomics identified changes in phosphatidylethanolamine, long-chain fatty acids, phosphatidylcholine and cholesterol esters, which differed after simvastatin (80 mg/day) compared with atorvastatin (40 mg/day) for 8 weeks in a placebo-controlled study. These drug-specific changes correlated with muscle expression of arachadonate 5-lipoxygenase-activating protein, involved in pro-inflammatory pathways. The authors postulated that the metabolomic approach may provide a sensitive marker of statin-induced changes in muscle and could be used to identify patients at risk of myopathy. The availability of a placebo control group is certainly a strength of the study. However, the study included a small number of participants (37 in total) and results were not corrected for false discovery rate (FDR). Moreover, the study did not include any participants with previous myopathy on a statin and a study in this population would be valuable. Even if some markers are eventually validated as useful predictors of myopathy, the health economic benefits of metabolite measurement would need to be established. Nevertheless, this is an interesting area of future research and may be applicable, in future, to high-risk individuals such as those with impaired hepatic or renal function, those on concomitant drug therapy known to increase the risk of myopathy or those with a family history of statin intolerance or muscle disease.^83^ Clearly, for other drugs/interventions where immediate optimal therapy is more critical, or where risk/benefit considerations are more complicated, pharmacometabolomic-guided therapy may be more valuable.

## Pragmatic limitations in applying metabolomics to large intervention studies and routine clinical use

### Analytical challenges

There are many challenges in metabolomics and lipidomics (Table 1).^10^^,^^85^ Most result from the complex mixture of metabolites found in biological samples such as serum and urine. Metabolites have a diverse range of physiochemical properties; therefore no single method can detect all of them.^21^ There is a limited ability to detect low concentration metabolites due to limitations in sensitivity, particularly with ^1^H-NMR metabolomics, narrowing the detectable dynamic range (ratio of highest versus lowest concentration: e.g. pM to mM). Problems with metabolite identification are common, partly due to limited availability of standards needed to confirm metabolite identity. Absolute quantitation, particularly in untargeted MS methods, is also problematic, again partially due to limited availability of stable isotope-labelled standards needed for quantification by MS due to variability in ion suppression and instrument stability. Inter-batch variation is an issue in MS metabolomics of studies with a large number of samples.^86^ Artefactual results due to variation in sample collection, storage and preparation are also known to be a problem for both metabolomics and routine clinical chemistry methods.^5^ High cost can be an issue, in particular for MS-based studies, and this often means smaller sample sizes with reduced power are often used, limiting the information gained.^85^ The benefits and limitations of MS and ^1^H-NMR metabolomics have been highlighted in a number of studies, and are generally described as complementary.^21^^,^^87^^,^^88^

Some of these problems can be overcome, and considerable effort has gone into optimizing available methods. Primarily, the analytical platform that naturally best meets the requirements to answer a specific research question should be chosen. Decisions on which analytical technique to use will often be pragmatic, based on instrument availability, costs, sample volumes or the need for absolute quantitation, high sensitivity, high throughput or improved coverage.^21^^,^^89^^,^^90^ Problems resulting from lack of coverage of the metabolome can be minimized by employing multiple analytical techniques in tandem as demonstrated in several of the studies described.^55^^,^^59^^,^^62–65^^,^^68^ Alternatively, multiple extraction techniques or columns and mobile phases can be employed in LC-MS. However, this can become costly and time consuming.^89^ Additionally, the drug of interest may direct the analytical technique chosen, for example the mechanism of action of lipid-lowering therapies may be more usefully probed using lipidomic methods rather than methods optimized for polar metabolites. However, the bile acid and simvastatin example demonstrates that this is not always the case.^62^^,^^64^ Problems with identification will be improved as spectral databases such as MassBank and the Human Metabolome Database (HMDB) continue to grow.^91^^,^^92^ Metabolites can also be identified by following up studies with more detailed methods such as two-dimensional NMR or high mass accuracy MS with fragmentation (MS^n^).^40^^,^^89^ Methods are emerging which allow absolute quantitation of a large number of metabolites by MS by including large numbers of labelled internal standards [Biocrates (current maxmum 180 metabolites and lipids) and Mass Isotopomer Ratio Analysis of U-^13^C-Labeled Extracts (MIRACLE)]^93^^,^^94^ Inter-batch variation in MS can be corrected for statistically using pooled quality control samples and statistical normalization methods,^9^^,^^86^ although this approach is only satisfactory for research (not clinical) purposes. Artefacts resulting from variation in sample handling can be minimized by the implementation of strict standard operating procedures.^5^ Advances in automation (robotic sample preparation, handling and delivery as well as automated data processing) are allowing metabolomics to be used in large-scale studies.^91^ This has contributed to the initially high costs associated with metabolomics being reduced.^91^ Costs can be further reduced by choosing high-throughput methods, for example, the ^1^H-NMR method described by Soininen *et al.*, 2015, which has been used in a large number of observational studies.^42^ However, it should be noted that this method quantifies only 18 metabolites due to limited sensitivity of ^1^H-NMR, the other metabolic measures being lipoprotein and lipid measures.^21^^,^^42^ Other methods that have been used in large-scale observational studies include the Metabolon method.^95^ This method is comparatively expensive and provides only relative (not absolute) quantification.^95^

Accepting that every technique, including those in routine biochemistry, has its limitations, we expect that many of these issues will not necessarily be overcome but minimized. There will always be trade-off between sensitivity, metabolite coverage and precision.^89^

### Interpretative challenges

There are multiple challenges in the interpretation of metabolomics studies (Table 1). There is the clear potential risk of false-positives and over-fitting models, particularly if there are a large number of ‘metabolite measures’ in relation to sample size.^96^ Another statistical issue relates to the analysis of multiple biomarkers in data that are highly correlated, and avoiding co-linearity in prediction models.

False-positive results can be minimized by appropriate statistical correction for multiple comparisons and by validation of models.^91^^,^^96^^,^^97^ Over-fitting of models can be reduced by cross-validation of the model (using an excluded test set) or, preferably, by validation in an independent external dataset (a separate follow-up study analysed separately).^91^^,^^98^ Co-linearity issues can been addressed using multivariate methods, such as principal component analysis (PCA) and orthogonal partial least squares discriminant analysis (OPLS-DA).^99^ However, such methods are not always appropriate, depending on the research aims.^97^^,^^98^ Highly correlated metabolites can also be removed from iterative models.^9^ More complicated statistical strategies for dealing with ’omic data (such as elastic net) have been reviewed.^100^

As clinical trial samples are very valuable and limited, careful use is essential. Most importantly, the studies need to be backed up by relevant clinical or mechanistic questions to avoid generating a series of results that, although of academic interest, do not advance clinical utility or provide mechanistic insights. This can be achieved by interdisciplinary work.^22^

Many of these obstacles (both analytical and interpretative) are not unique to metabolomics, and also apply to emerging chemistry-based markers, proteomics, transcriptomics and genomics.^19^^,^^79^^,^^101^ Therefore, both analytical technologies and computational methods are constantly advancing and evolving. It should be stressed that despite these limitations, studies have shown the power of metabolomics in mechanism of action studies to generate novel hypothesis.^10^

## How can real gains be made?

The issues described above suggest that considerable work is required to better test the clinical utility of metabolomics in clinical trial datasets. We now provide a list of recommendations, in part mirroring a roadmap for implementation of proteomic biomarkers which is equally valid for metabolic biomarkers.^22^

- Analysis of existing larger trials as well as trials including a wide range of age groups, ethnicities and other characteristics, or at least representing the population the drugs are expected to be prescribed for, rather than only healthy volunteers.
- Use of data from existing trials with hard endpoints and not just surrogate endpoints. This allows testing whether pharmacometabolomics can predict the out-comes that really matter in clinical practice.
- Use of data from trials with a control arm: either placebo-controlled or a new therapy compared with the current gold-standard pharmacotherapy.
- Comparison of predictive abilities of potential new biomarkers versus gold-standard conventional biomarkers. Such an approach enables proper assessment of incremental utility gained by novel biomarkers over and above established tests.
- Standardization of methods, including collection and storage of samples, to lessen the effect of measurement artefacts on data.
- Implementation of fully validated methodology with absolute quantification, batch correction and confident identification where possible, to yield data that can be trusted, compared between studies and interpreted (biologically) more easily.
- Whenever possible, validation of any novel findings in an independent cohort to give added confidence that results are robust and generalizable.
- Economic analysis to estimate whether any incremental benefits justify the increase in costs or to determine realistic prices to enable clinical consideration.
- Dissemination, communication and working with a range of stakeholders: scientists, patients, clinicians, clinical scientists, regulatory bodies, expert committees, statisticians, health economists, pharmaceutical/biotechnology companies and biobanks. This ensures studies are relevant, well designed and conducted, and useful.

## Conclusion

Metabolomics has been applied to biobanked samples from clinical trials in recent years, but selected studies have typically been of a small scale and focused on surrogate outcomes, with the consequence that published evidence has indicated at best only modest potential. If such findings can be extended and properly validated, there remains a potential for metabolomics to aid better tailoring of drugs to patients (beyond those enrolled in clinical trials). Accordingly, pharmacometabolomics has been described as a ‘potential gateway’ to stratified medicine.^12^^,^^13^ However, the usefulness of pharmacometabolomics in clinical trials will likely vary from drug to drug, depending on how well safety or efficacy can be predicted from simpler tests, what other options are currently available and the cost of sample analysis.^12^^,^^102^ Current evidence in this area remains exciting but is largely at the hypothesis-generating or proof-of-concept stage. As such, further assessment of its use is required in a larger number of robust studies or trials.

For real advances to be made, investigators with metabolomics expertise need to work with clinicians and statisticians to: (i) develop rigorous experimental study designs; (ii) to identify the best biobanks to exploit; and (iii) to carefully outline the key clinical questions that could be usefully addressed by metabolomics at the very beginning of the project.

## Funding

N.R. is supported by the UPBEAT RCT mother-child study; stratifying and treating obese pregnant women to prevent adverse pregnancy, perinatal and longer term outcomes: Medical Research Council (MRC) (MR/L002477/1). Our recent work in ^1^H-NMR metabolomics is supported by: (i) the Chief Scientist Office, Scotland (CZB/4/613); (ii) the Wellcome Trust Institutional Strategic Support Fund (ISSF) (WT097821MF); (iii) the European Federation of Pharmaceutical Industries Associations (EFPIA) Innovative Medicines Initiative Joint Undertaking (EMIF) (grant number 115372); (iv) Chest, Heart & Stroke, Scotland (R13/A149); and (v) the European commission, under the Health Cooperation Work Programme of the 7th Framework Programme (grant number 305507) under Heart ‘OMics’ in AGEing (HOMAGE).

**Conflict of interest** **:** N.S. has consulted for AstraZeneca, Bristol-Myers Squibb, Amgen, Sanofi and Boehringer.
